# Artificial intelligence used to diagnose osteoporosis from risk factors in clinical data and proposing sports protocols

**DOI:** 10.1038/s41598-022-23184-y

**Published:** 2022-10-31

**Authors:** Leila Fasihi, Bakhtyar Tartibian, Rasoul Eslami, Hossein Fasihi

**Affiliations:** 1 Department of Physical Education and Sport Sciences, Faculty of Humanities, Tarbiat Modares University, Tehran, Iran; 2 Department of Exercise Physiology, Faculty of Physical Education and Sport Sciences, Allameh Tabataba’i University, Tehran, Iran; 3 School of Railway Engineering, Iran University of Science and Technology (IUST), Tehran, Iran

**Keywords:** Physiology, Diseases, Health care, Medical research, Rheumatology, Risk factors

## Abstract

Osteoporosis (OP) is characterized by diminished bone mass and deteriorating bone structure that increases the chance of fractures in the spine, hips, and wrists. In this paper, a novel data processing method of artificial intelligence (AI) is used for evaluating, predicting, and classifying OP risk factors in clinical data of men and women separately. Additionally, artificial intelligence was used to suggest the most appropriate sports programs for treatment. Data was obtained from dual-energy x-ray absorption scanning center of Ayatollah Kashani, Milad, and Khatam al-Anbia hospitals in Tehran, Iran. The subjects included 1224 men and women. Models were developed using decision tree, random forest (RF), k-nearest neighbor, support vector machine, gradient boosting (GB), Extra trees, Ada Boost (AB), and artificial neural network multilayer perceptron analysis to predict osteoporosis and to recommend sports programs. Data was divided into training (80%) and test dataset (20%). The results were obtained on a 20% test dataset. Area under receiver operating characteristic curve (AUROC) was used to compare the performance of the models. To predict healthy individuals, osteopenia and osteoporosis, the FR algorithm with AUROC 0.91 performed best in men and the GB algorithm with AUROC 0.95 performed best in women compared to other classification algorithms. Prediction of RF algorithm in women and men with AUROC 0.96 and 0.99, respectively, showed the highest performance in diagnosing the type of exercise for healthy individuals and those with osteopenia and OP. Eight AI algorithms were developed and compared to accurately predict osteoporosis risk factors and classify individuals into three categories: healthy, osteopenia, and OP. In addition, the AI algorithms were developed to recommend the most appropriate sports programs as part of treatment. Applying the AI algorithms in a clinical setting could help primary care providers classify patients with osteoporosis and improve treatment by recommending appropriate exercise programs.

## Introduction

Osteoporosis (OP) is a common metabolic systemic bone disease characterized by increased bone fragility, low bone mass, and a high risk of fractures leading to falls and decreased bone mineral density (BMD)^1^. OP is considered a great public health problem and the most common metabolic bone disease as it causes more than 8.9 million fractures per year, resulting in one fracture every three seconds and affecting more than 200 million people worldwide^2^. To increase physicians' awareness of asymptomatic osteoporosis and to identify at-risk patients, it is crucial to understand the risk factors and appropriately diagnose the disease^3^. Several factors such as gender, age, body mass index (BMI), height, low body weight, adequate levels of physical activity, poor nutritional status, family history, calcium, and vitamin D intake, back pain, and other endocrine and cardiometabolic factors are associated with osteoporosis and very important in diagnosing it during lifetime^4,5^. Although the most widely used clinical tool for measuring BMD and assessing bone strength is laboratory dual-energy X-ray absorption (DXA), the availability of DXA is very limited^6^ and does not indicate bone quality^7^. Therefore, appropriate methods for screening, diagnosis and monitoring of these patients are needed^8^. Many researchers have also aimed to develop predictive models using risk factors for the screening of osteoporosis^9,10^.

In recent years, other than traditional modeling, classification algorithms have gained popularity because of their ability to detect more complex relationships between input and output features and flexible modeling^11,12^. Classification algorithms, using large volumes of data, make new information and relationships embedded in large and complex datasets visible through inferring and learning new patterns and relationships^13^. At present, the machine learning approach is not sufficient to predict osteoporosis with a larger data set in men and women and requires further study. Hence, the first goal of the present study was to determine osteoporosis risk factors in clinical data comprising of physical characteristics, personal and medical history, and laboratory tests in men and women. By using classification algorithms in clinical practice as a screening tool, both physicians and patients would be more aware of osteoporosis risk factors and take more preventive measures in the early stages of the disease to avoid adverse outcomes.

Drug treatments, fall precautions, and lifestyle changes suggested to patients with osteoporosis have led to a 21–66 percent reduction in fracture risk^14^. Despite the availability of effective anabolic and anti-absorption drugs, osteoporosis and related fractures remain an unsolvable problem, forcing health organizations to recently launch a “Call to Action” to address the crisis in osteoporosis treatment^15^. Exaggerated concerns regarding the side effects of some medications have resulted in the use of exercise to prevent osteoporosis^16^.

Physical activity (PA) is recommended as a safe and low-cost non-pharmacological intervention strategy to change bone risk factors and maintain musculoskeletal health^17^. It has been shown that the mechanical load resulting from PA increases muscle mass, creates mechanical stress on the skeleton, and increases osteoblast activity^18^. Various physical activities are effective in preventing and treating OP and AI is effective in data classification and quick access to results^19,20^. Due to the beneficial effects of PA in the prevention of osteoporosis, physician and patient accessibility to appropriate and effective sports activities is essential^21^. The second goal of this study was to develop artificial intelligence to propose appropriate exercise protocols for OP patients' improvement.

## Materials and methods

### Ethics

The present study was a prospective cohort design approved by the Research Ethics Committee of Allameh Tabataba’i University with ID: IR.ATU.REC.1399.038. Subjects provided written informed consent to participate in the study and all research was conducted according to the relevant instructions. In order to protect the privacy of these clinical data, all ethical principles of patients’ rights have been heed and participants’ names were not mentioned.

### Study participants

A total of 1224 patients of Ayatollah Kashani, Milad, and Khatam al-Anbia hospitals (Tehran, Iran) in the period 2019–2021 were included in the study. The inclusion criteria comprised of the age range of 35–85 years, with clinical risk factors related to osteoporosis. The sample size using G-Power software with effect size of 0.15, test power of 0.89 and error value of 0.05 was 368 participants. For greater assurance in this study, the sample size was increased to 1224 participants. Patients were separated into women (n = 754) and men groups (n = 470).

### Outcome T-score measurements

A dual-energy X-ray absorptiometry (QDR 4500; Hologic, Bedford, MA, USA) was used to measure T-score and BMD in the femoral neck, lumbar spine (L1–L4) and total femur. The most important aspect of DXA interpretation is estimating a patient’s risk of developing an osteoporosis-related fracture. The DXA information includes the BMD, T-score, and Z-score. A T-score indicates the number of standard deviations below or above the BMD average, expressed in grams per square centimeter. This BMD value is compared to the BMD of a population of young adults of the same gender, and several standard deviations (SD) near each of these values. The difference between the mass of the population of young adults of the same gender and the current bone mass of the patient examined is called T-score^22^. According to World Health Organization (WHO), healthy or normal is diagnosed when the T-score is greater than − 1.0 SD, osteopenia is diagnosed when the T-score is between − 1.5 and − 2.5, and osteoporosis is diagnosed when the T-score is more than − 2.5 SD^23^.

### Feature selection and assessment of covariates

The present study was a prospective cohort design. In this study, by reviewing similar articles and consulting with a specialist physician, and distributing and collecting relevant questionnaires from the subjects, the most significant clinical factors of osteoporosis were prepared. In addition, in a review of relevant books and articles, clinical factors related to osteoporosis were extracted^24^. Finally, 19 input features for women and 17 input features for men (excluding menopause and menopausal age) were selected as the input of the algorithms.

The input features applied to the algorithms included: age, height, weight, body mass index (BMI), curvature of the spine, family history of osteoporosis, Vitamin D intake, physical activity, back pain, bone fracture, parathyroid disease, history of smoking, and serum levels of calcium, phosphorus, vitamin D, and alkaline phosphatase (ALP). 0 and 1 were used for questions with yes and no answers (0 meaning no, 1 meaning yes). A questionnaire was used to diagnose parathyroid disease. A family history of osteoporosis was considered when at least one first-degree relative was diagnosed with osteoporosis. A physical activity questionnaire was used to assess the level of subject physical activity. Smoking was classified as "never" and "now". For women, menopausal status and menopausal age were also considered. For analysis, the data set in Excel format was transferred to Python version 3.10 (Python Software Foundation, Wilmington, DE, USA).

Subjects had to meet the following criteria: to be aged between 35 and 85 years, male/female gender, BMI of 18 to 40, T-score of 2 to − 3, absence of metabolic disease, rheumatoid arthritis and bone cancer, neither taking corticosteroids nor exercising regularly in the past year. Subjects also had to demonstrate they had no secondary illness, history of surgery or injury, or any physical problems. Table 1 shows the description of the dataset features.

**Table 1:** Description of the dataset features.

Sample number	Feature	Units	Range
1	Age	Years	35–85
2	Height	Cm	140–199
3	Weight	Kg	30–120
4	BMI	Kg/m^2^	18–40
5	The curvature of the spine	Yes (1), no (0)	0.1
6	Family history	Yes (1), no (0)	0.1
7	Vitamin D intake	Yes (1), no (0)	0.1
8	Physical activity (PA)	Yes (1), no (0)	0.1
9	Ca intake	Yes (1), no (0)	0.1
10	Back pain	Yes (1), no (0)	0.1
11	Bone fracture	Yes (1), no (0)	0.1
12	Parathyroid disease	Yes (1), no (0)	0.1
13	Smoking	Yes (1), no (0)	0.1
14	Serum calcium	mg/dL	8–10.5
15	Serum phosphorus	mg/dL	4–6
16	Serum vitamin D	mg/dL	7–50
17	Serum ALP	U/L	70–430
18	menopause	Yes (1), no (0)	0.1
19	menopausal age	Years	45–55
Label	Healthy (1), Osteopenia (2), Osteoporosis (3)		
	Sport protocols (1–9)		

### Data analysis to predict Healthy people, osteopenia, osteoporosis and proposing sports protocols

Candidate algorithms in this study included DT, RF, KNN, SVM, GB, ET, AB and ANN. Due to differences in input characteristics, baseline characteristics, and the prevalence of positive predictions, the models were trained separately in men and women Experimental data sets were applied to different models to obtain predictive probabilities of osteopenia, osteoporosis, and disease probability or absence in each model, and according to age and health status in both groups of men and women using the AI methods and to provide suitable sports protocol. For this purpose, all sports therapy protocols collected in scientific articles and classified based on various effective parameters (such as age, gender and health status). Finally, to predict and prescribe the type of exercise, appropriate algorithms were trained.

The model was trained by predicting three groups, which included healthy, osteopenia, and osteoporosis. During the training process, the goal was to predict the multivariate model (1, 2, 3). In the testing process, the prediction results were marked “1” for the healthy group, “2 and 3” for the osteopenia and osteoporosis groups, respectively. For classifying sports, several binary variables were presented (1, 2, 3, 4, 5, 6, 7, 8, 9) shown separately for men and women in Tables 4 and 5.

**Figure 1:**
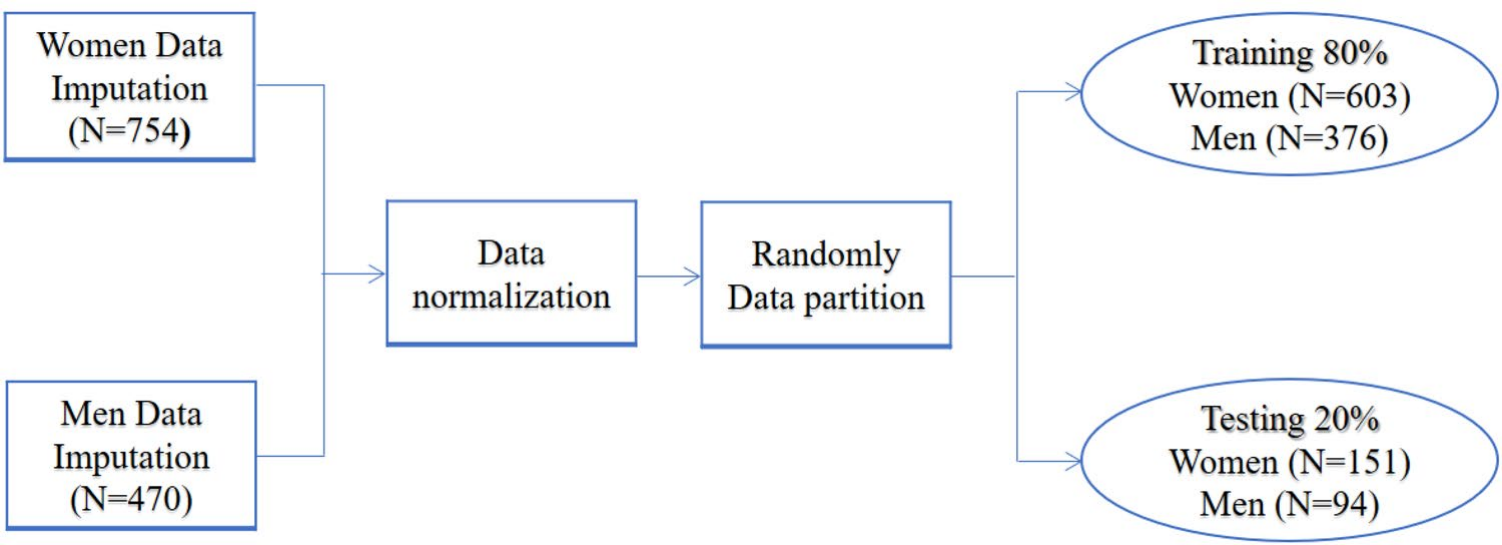
Data processing.

The separate datasets for men and women were divided into training and testing datasets with an 80:20 split. During each training section of models, used a 20% test dataset to experiment the performance of the models. The data was analyzed, and the algorithm with best performance identified. This resulted in 376 men in training and 94 men in testing datasets, and 603 women in training and 151 women in testing datasets. Figure 1 illustrates data processing in the present study.

DT is the denotative representation of a decision-making process. DT in AI is used to arrive at conclusions based on the data available from decisions made in the past^25^. The random forest (RF) is comprised of a large number of individual decision trees that act as a collection. Each single tree in the random forest publishes a class prediction, which uses a randomly drawn subsample and a random subset of the available features for each splitting decision^25^. The KNN algorithm uses 'feature similarity' to predict the values of any new data points. This means that the new point is assigned a value based on how closely it resembles the points in the training set^26^. SVM falls into the category of supervised learning which is useful for solving classification problems. This method creates the best decision boundary for dividing the next n space into different classes to place the new data points in the appropriate class category. SVM always selects strong vectors to create hyperplanes. These extremes are called backup vectors. The dimensions of the hyperplane are determined by the number of data set features^27^. GB combines the predictions from multiple decision trees to generate the final predictions. The ET algorithm operates by creating a large number of unpruned decision trees from the training dataset. Predictions are made by averaging the prediction of the decision trees in the case of classification. AB functions by putting more weight on difficult to classify instances and less on those already handled well^28^. A multilayer perceptron (MLP) is a fully connected class of feedforward artificial neural network (ANN). The term MLP is used ambiguously, sometimes loosely to mean any feedforward ANN, sometimes strictly to refer to networks composed of multiple layers of perceptrons. MLP is a feedforward artificial neural network that generates a set of outputs from a set of inputs. An MLP is characterized by several layers of input nodes connected as a directed graph between the input and output layers^29^. The process and the final selected hyperparameters for each model are presented in Table 2.

**Table 2:** Selection of hyperparameters for the DT, RF, KNN, SVM, GB, ET, AB and ANN models.

Models	Selected hyperparameter for the men model to predict healthy, osteopenia, and OP	Selected hyperparameter for the women model to predict healthy, osteopenia, and OP	Selected hyperparameter for the men model to propose sports protocols	Selected hyperparameter for the women model to propose sports protocols
DT	max_depth = 4, max_leaf_nodes = 6	max_depth = 5, max_leaf_nodes = 7	max_depth = 3, max_leaf_nodes = 7	max_depth = 6, max_leaf_nodes = 12
RF	max_depth = 7, random_state = 3, n_estimators = 19	max_depth = 10, random_state = 17, n_estimators = 9	max_depth = 7, random_state = 14, n_estimators = 13	max_depth = 13, random_state = 7, n_estimators = 16
KNN	n_neighbors = 3	n_neighbors = 10	n_neighbors = 2	n_neighbors = 4
SVM	C = 17, kernel = 'rbf', gamma = 'scale', coef0 = 0.0, shrinking = True, probability = True, tol = 0.001, class_weight = None, verbose = False, decision_function_shape = 'ovr', break_ties = False, random_state = 0	C = 28, kernel = 'rbf', gamma = 'scale', coef0 = 0.0, shrinking = True, probability = True, tol = 0.001, class_weight = None, verbose = False, decision_function_shape = 'ovr', break_ties = False, random_state = 0	C = 15, kernel = 'rbf', gamma = 'scale', coef0 = 0.0, shrinking = True, probability = True, tol = 0.001, class_weight = None, verbose = False, decision_function_shape = 'ovr', break_ties = False, random_state = 0	C = 37, kernel = 'rbf', gamma = 'scale', coef0 = 0.0, shrinking = True, probability = True, tol = 0.001, class_weight = None, verbose = False, decision_function_shape = 'ovr', break_ties = False, random_state = 0
GB	max_depth = 22, min_samples_split = 8, min_samples_leaf = 14, max_features = 2	max_depth = 3, min_samples_split = 9, min_samples_leaf = 6, max_features = 5	max_depth = 13, min_samples_split = 14, min_samples_leaf = 12	max_depth = 6, min_samples_split = 10, min_samples_leaf = 7
ET	max_depth = 8, min_samples_split = 9, min_samples_leaf = 3, max_features = 11	max_depth = 15, min_samples_split = 3, min_samples_leaf = 6, max_features = 15	max_depth = 11, min_samples_split = 2, min_samples_leaf = 1, max_features = 8	max_depth = 22, min_samples_split = 2, min_samples_leaf = 2, max_features = 15
AB	n_estimators = 10, base_estimator = base, learning_rate = 0.1	n_estimators = 4, base_estimator = base, learning_rate = 0.1	n_estimators = 29, base_estimator = base, learning_rate = 0.1	n_estimators = 13, base_estimator = base, learning_rate = 0.1
ANN	activation = 'tanh', alpha = 0.0001, batch_size = 'auto', hidden_layer_sizes = 35, learning_rate = 'constant', max_iter = 470	activation = 'tanh', alpha = 0.0001, batch_size = 'auto', hidden_layer_sizes = 25, learning_rate = 'constant', max_iter = 754	activation = 'tanh', alpha = 0.0001, batch_size = 'auto', hidden_layer_sizes = 30, learning_rate = 'constant', max_iter = 470	activation = 'tanh', alpha = 0.0001, batch_size = 'auto', hidden_layer_sizes = 27, learning_rate = 'constant', max_iter = 754

Various combinations of the number of layers (1 or 40 hidden layers), learning rate (from 0.01 to 0.0001) were investigated in the ANN model for hyperparameter tuning. For the DT model, the depth and leaf parameters and SVM, the kernel type (linear, polynomial, or radial basis function), regularization parameter C (from 2^–2^ to 2^9^) and for the RF model, the max depth (from 3 to 13), random state (from 3 to 17), were examined; for the KNN model, the number of neighbors (from 1 to 10) were tested. All models were created with a balanced class weight. Randomization for the split of validation dataset and the training process was repeated numerous times for each hyperparameter set. The area under the receiver operating characteristic curve (AUROC) was estimated for the test dataset in each training process, and the mean values of AUROC were compared. The Area Under the Curve (AUC) is a measure of the capability of a classifier to distinguish between classes and is utilized as a summary of the ROC curve. The greater the AUC, the better the performance of the model at discerning positive and negative classes. Other values were calculated in the performance of the algorithms for the data set in each training process including accuracy, precision, sensitivity, specificity, and F-score. Accuracy is an evaluation criteria of classification models and is a fraction of the predictions that the model has made accurately (Eq. 1). Precision is a metric calculating the percentage of correct predictions for the positive class. Maximizing precision will minimize the false-positive errors, whereas maximizing recall will minimize the false-negative errors (Eq. 2). Sensitivity (also known as recall) estimates the percentage of correct predictions for the positive class out of all possible positive predictions made (Eq. 3). Specificity calculates the proportion of true negatives correctly identified by the model (Eq. 4). The F-measure, also named the F-score, is used to verify the accuracy of a model in a data set. F-score is widely used in evaluation of information retrieval systems such as search engines and many types of machine learning models. In addition, the F-measure is a configurable single-score metric for evaluating a binary classification model based on the predictions made for the positive class (Eq. 5). The F-measure is estimated using sensitivity and precision^30^. The following criteria were calculated to evaluate the performance of the predicted models.

1 $${\text{Accuracy}} = \left( {{\text{true positives}} + {\text{true negatives}}} \right)/\left( {\text{total examples}} \right)$$

2 $${\text{Precision}} = \left( {\text{true positives}} \right)/\left( {{\text{true positives}} + {\text{false positives}}} \right)$$

3 $${\text{Sensitivity}} = \left( {\text{true positives}} \right)/\left( {{\text{true positives}} + {\text{false negatives}}} \right)$$

4 $${\text{Specificity}} = \left( {\text{true negative}} \right)/\left( {{\text{true negative}} + {\text{false positive}}} \right)$$

5 $${\text{F-score}} = ({2} \times {\text{precision}} \times {\text{recall}})/\left( {{\text{precision}} + {\text{recall}}} \right)$$

### Ethics approval and consent to participate

This study was approved by the Ethics Committee of Allameh Tabataba’i University with research ID Code: IR.ATU.REC.1399.038. Subjects provided written informed consent to participate in this study and all research was conducted according to the relevant instructions.

## Results

1224 subjects participated in the study (men: n = 470 and women: n = 754). The results of DXA showed that 72 men and 149 women had osteoporosis, 233 men and 427 women had osteopenia, and 165 men and 178 women were healthy. The demographic information is summarized in Table 3.

**Table 3:** Demographic information of male and female participants.

Characteristic	Mean and standard deviation	
	Men (n = 470)	Women (n = 754)
Age (years)	55.37 ± 13.67	57.70 ± 9.67
Height (cm)	171.87 ± 8.32	159.45 ± 6.23
Weight (kg)	77.79 ± 12.62	72.92 ± 12.30
BMI (kg/m^2^)	26.37 ± 4.17	28.70 ± 4.78
Serum calcium (mg/dl)	8.88 ± 0.61	8.98 ± 0.48
Serum phosphorus (mg/dl)	4.08 ± 0.55	3.81 ± 0.51
Serum Vitamin D (mg/dl)	25.22 ± 9.19	21.92 ± 7.81
Serum ALP (U/L)	158.83 ± 54.53	177.35 ± 75.71
Total femur BMD (g/cm^2^)	1.05 ± 0.14	0.922 ± 0.14
Femoral neck BMD (g/cm^2^)	0.899 ± 0.15	0.779 ± 0.12
Lumbar spine BMD (g/cm^2^)	1.11 ± 0.19	1.01 ± 0.19
Age of menopausal (years)	–	50.77 ± 1.48

*Step 1* At this stage, 8 classification algorithms (DT, RF, KNN, SVM, GB, ET, AB, ANN) were used to predict and divide people into three categories: healthy, osteopenia and osteoporosis.

19 features for women and 17 features for men (excluding menopause and menopausal age) were selected as input characteristics of these algorithms with three objectives of healthy (T-score > − 1.0), osteopenia (T-score between − 1.5 and − 2.5) and osteoporosis (T-score ≤ − 2.5).

**Figure 2:**
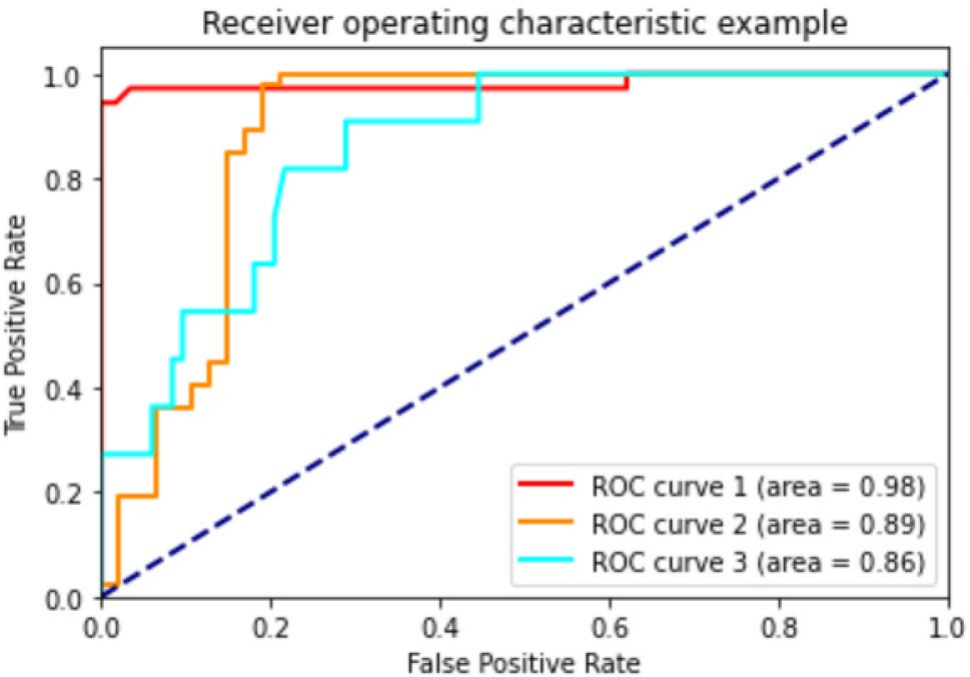
AUROC in RF algorithm for predicting healthy, osteopenia, and osteoporosis in men.

**Figure 3:**
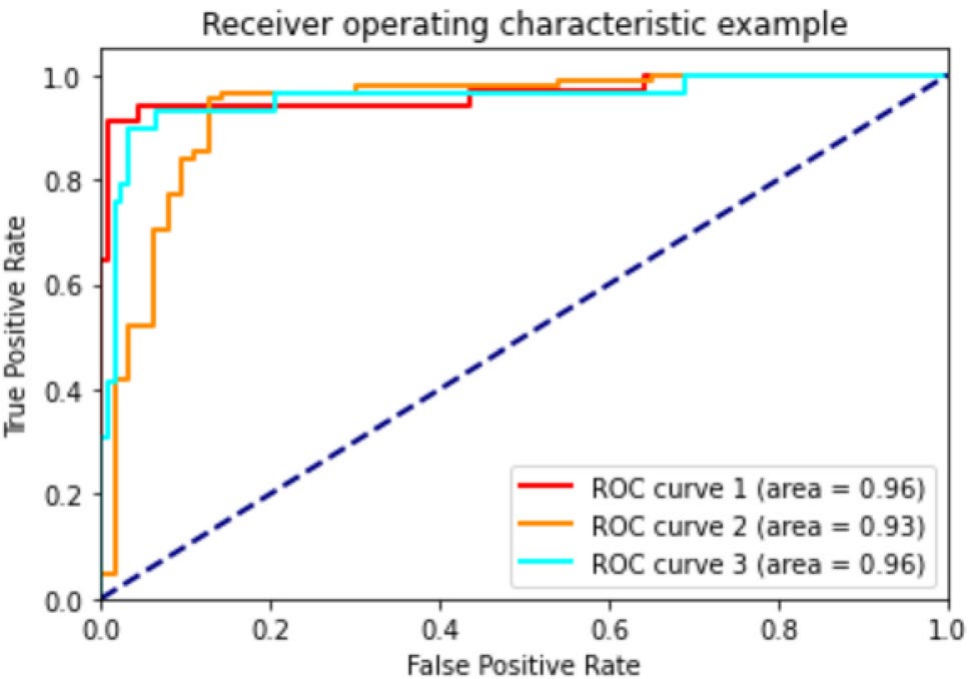
AUROC in GB algorithm for predicting healthy, osteopenia, and osteoporosis in women.

According to the results of Table 3 machine learning models achieved 73–85% accuracy, 71–90% precision, 64–76% sensitivity and 81–89% specificity in classifying men participants into the three categories of healthy, osteopenia, and osteoporosis. To predict healthy, osteopenia, and osteoporosis in women, machine learning models achieved an accuracy of 75–93%, a precision of 74–93%, a sensitivity of 75–91% and a specificity of 82–95%. In addition, the AUROC between machine learning models were compared. The RF algorithm showed the highest performance in the men group with AUROC 0.91, and in the women's group, the GB algorithm with AUROC 0.95 performed best. The ROC curves of different models of machine learning in men and women are presented in Figs. 2 and 3 (Table 4).

**Table 4:** Comparison of different classification algorithms in both men and women to predict healthy individuals, and individuals with osteopenia and OP.

	Models	AUROC	Accuracy	Precision	Sensitivity	specificity	F-score
Men	DT	0.84	0.81	0.89	0.70	0.86	0.71
	RF	0.91	0.85	0.92	0.76	0.89	0.79
	KNN	0.86	0.73	0.71	0.70	0.83	0.69
	SVM	0.84	0.74	0.83	0.64	0.82	0.65
	GB	0.89	0.83	0.90	0.75	0.88	0.77
	ET	0.88	0.80	0.88	0.70	0.86	0.73
	AB	0.84	0.81	0.89	0.70	0.86	0.71
	ANN	0.90	0.73	0.77	0.64	0.81	0.65
Women	DT	0.91	0.91	0.93	0.87	0.93	0.90
	RF	0.93	0.92	0.91	0.90	0.95	0.91
	KNN	0.87	0.79	0.80	0.76	0.86	0.77
	SVM	0.86	0.82	0.83	0.77	0.87	0.79
	GB	0.95	0.93	0.93	0.91	0.95	0.92
	ET	0.94	0.91	0.93	0.87	0.93	0.90
	AB	0.87	0.91	0.93	0.87	0.93	0.90
	ANN	0.84	0.75	0.74	0.75	0.82	0.76

*Step 2* In the second stage, sports protocols were proposed to healthy individuals and those with osteopenia and osteoporosis using 8 classification algorithms and 9 labels to predict exercise protocols for men and women separately.

Table 5 shows the recommended exercises for men in the three categories of health status. Numbers 1, 2, and 3 denote healthy men’s (T-score > − 1.0) age ranges of 35–50, 51–65 and 66–85, respectively; numbers 4, 5 and 6 correspond to the same age ranges of 35–50, 51–65 and 66–85 in men with osteopenia (T-score between − 1.5 and − 2.5); and finally the last category of men with osteoporosis (T-score ≤ − 2.5) is represented by numbers 7, 8 and 9 with respective age ranges of 35–50, 51–65 and 66–85.

**Table 5:** Recommended exercises for men in three categories of healthy, osteopenia and OP.

Number	age	Length	Intervention	T-score range
1	35–50	6 months 2–3/week	Resistance training with intensity of 80–75% 1RM, aerobic training with 75% intensity of reserve heart rate, HIIT training, BFR moderate-intensity resistance training, HIT training, jumping training	Healthy (T-score > − 1.0)
2	51–65	8 months 2–3/week	Resistance training with 65% intensity 1RM, balance training, brisk walking and reaction training, training with TRX at 5 Likert scale	
3	Over 66	12 months 2–3/week	Whole-body vibration, cycling, resistance training with 60% intensity 1RM, aerobic training with 60% intensity of heart rate reserve, balance exercises + flexibility + reaction	
4	35–50	12 months 2–3/week	Resistance training with 70% intensity 1RM, medium intensity aerobic training with up to 70% reserve heart rate, cycling, training with TRX at 4 Likert scales, agility and reaction speed training, HIIT training	Osteopenia (T-score − 1.5 to − 2.5)
5	51–65	12 months 2–3/week	Resistance training with 65% intensity 1RM Aerobic exercises with 65% intensity of heart rate reserve, balance exercises, brisk walking	
6	Over 66	12 months 2–3/week	Cycling, balance exercises, brisk walking, whole- body vibration, aerobic exercises with 60% reserve heart rate, and water training, resistance exercises with 60% 1RM intensity	
7	35–50	12 months 2–3/week	Medium intensity combined exercises (including walking, running, balance and flexibility), cycling, resistance training with 60% intensity 1RM aerobic training with 60% reserve heart rate, training with TRX at 3 Likert scale level	Osteoporosis (T-score ≤ − 2.5)
8	51–65	12 months 2–3/week	Water training with an average intensity of 55% of the reserve heart rate, resistance training with an intensity of 55% 1RM Aerobic training with an intensity of 60% of the reserve heart rate	
9	Over 66	12 months 2–3/week	Aerobic exercise with 50% reserve heart rate, water exercise with 50% reserve heart rate, gentle walking, whole-body vibration, balance and flexibility exercises), functional exercises	

*Within the examined range, different classification algorithms were used. *DT* Decision tree, *RF* Random forest, *KNN* k-nearest neighbor, *SVM* Support vector machine, *GB* Gradient boosting, *ET* Extra trees, *AB* Ada Boost, *ANN-MLP* Artificial neural network multilayer perceptron.

Table 6 indicates the recommended exercises for women in the three categories of health status. Numbers 1, 2, and 3 denote healthy women's (T-score > − 1.0) age ranges of 35–50, 51–65 and 66–85, respectively; numbers 4, 5 and 6 correspond to the same age ranges of 35–50, 51–65 and 66–85 in women with osteopenia (T-score between − 1.5 and − 2.5); and finally the last category of women with osteoporosis (T-score ≤ − 2.5) is represented by numbers 7, 8 and 9 with respective age ranges of 35–50, 51–65 and 66–85.

**Table 6:** Recommended exercises for women in the three categories of healthy, osteopenia and OP.

Number	age	Length	Intervention	T-score range
1	35–50	8 months 2–3/week	Agility training, HIIT, resistance training with the intensity of 80–75% 1RM, aerobic training with 75% intensity of heart rate reserve, HIT training, jumping training, training with TRX at 4 Likert scale level, reaction speed training	Healthy (T-score > − 1.0)
2	51–65	12 months 2–3/week	Water preparation exercises with 70% reserve heart rate, resistance exercises with 70% 1RM intensity, aerobic exercises with 70% reserve heart rate, cycling, fast walking	
3	Over 66	12 months 2–3/week	Water aerobic exercise with 65% reserve heart rate, balance and flexibility exercises + full body vibration, fast walking, exercises with TRX at 3 Likert scale	
4	35–50	12 months 2–3/week	Aerobics + Pilates + Whole Body Vibration, Aerobic Exercises in Water with 65% Reserve Heart Rate Intensity, Medium Intensity Combined Exercises (Including Running, Balance and Flexibility), 1RM 65% Resistance Exercises, 4 Likert Level TRX Exercises	Osteopenia (T-score − 1.5 to − 2.5)
5	51–65	12 months 2–3/week	Water training with 60% reserve heart rate, resistance training with 65% 1RM intensity, cycling, balance, and flexibility training	
6	Over 66	12 months 2–3/week	Exercise in water with 55% reserve heart rate, balance and flexibility exercises + whole-body vibration, brisk walking, exercises with TRX at level 2 Likert scale	
7	35–50	12 months 2–3/week	Pilates + Aerobics + whole-body vibration, aerobic exercise in the water with 60% reserve heart rate, combination exercises (including running, balance, and flexibility and 60% reserve exercise), resistance training with 60% RR intensity	Osteoporosis (T-score ≤ − 2.5)
8	51–65	12 months 2–3/week	Water training with 55% reserve heart rate, brisk walking, training with TRX at level 2 Likert scale, resistance training with 55% intensity 1RM	
9	Over 66	12 months 2–3/week	Hydrotherapy exercises with TRX level 1 Likert scale, brisk walking, functional exercises with 50% intensity of heart rate Save reaction exercises	

*Within the examined range, different classification algorithms were used. *DT* Decision tree, *RF* Random forest, *KNN* k-nearest neighbor, *SVM* Support vector machine, *GB* Gradient boosting, *ET* Extra trees, *AB* Ada Boost, *ANN-MLP* Artificial neural network multilayer perceptron.

In Table 7, the results of the men and women data classification algorithms for the proposed sports protocols are compared.

**Table 7:** Comparison of predictions of classification algorithms to recommend sports protocols between men and women groups.

	Models	AUROC	Accuracy	Precision	Sensitivity	specificity	F-score
Men	DT	0.86	0.78	0.51	0.56	0.96	0.52
	RF	0.89	0.84	0.70	0.67	0.97	0.64
	KNN	0.81	0.56	0.45	0.50	0.90	0.46
	SVM	0.82	0.70	0.56	0.52	0.94	0.52
	GB	0.95	0.84	0.78	0.69	0.87	0.70
	ET	0.94	0.82	0.73	0.68	0.96	0.67
	AB	0.96	0.82	0.75	0.67	0.96	0.67
	ANN	0.88	0.71	0.57	0.58	0.94	0.56
Women	DT	0.94	0.92	0.90	0.88	0.98	0.87
	RF	0.98	0.98	0.99	0.97	0.99	0.98
	KNN	0.86	0.65	0.50	0.49	0.92	0.48
	SVM	0.91	0.76	0.59	0.58	0.95	0.58
	GB	0.93	0.97	0.93	0.97	0.99	0.94
	ET	0.98	0.93	0.90	0.92	0.98	0.90
	AB	0.99	0.94	0.96	0.90	0.98	0.92
	ANN	0.89	0.53	0.45	0.37	0.89	0.34

**Figure 4:**
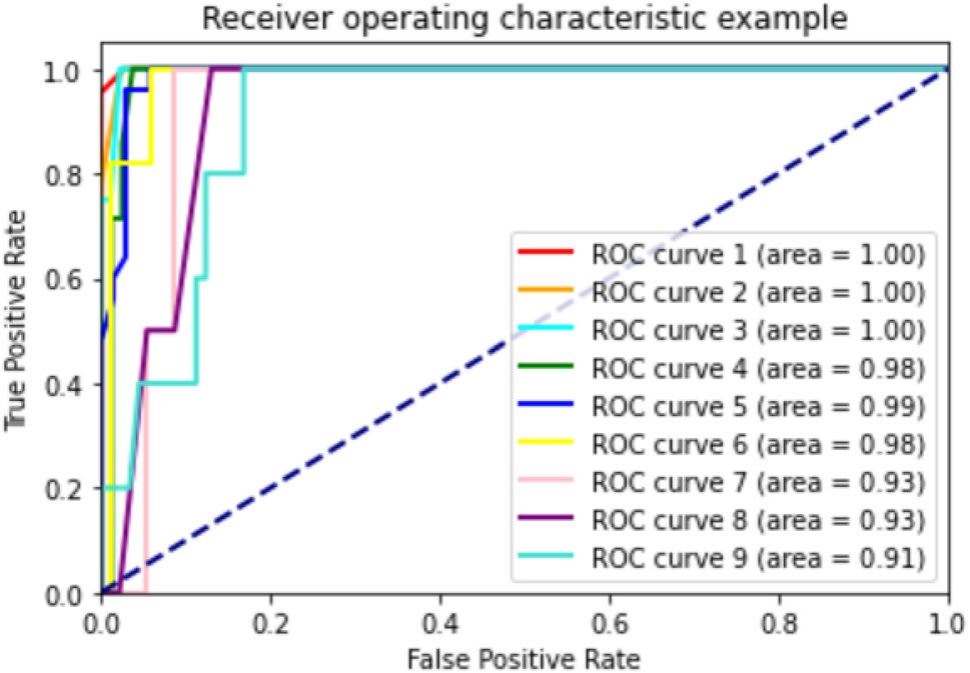
AUROC in RF algorithm for predicting exercise appropriate for healthy men and those with osteopenia and OP.

**Figure 5:**
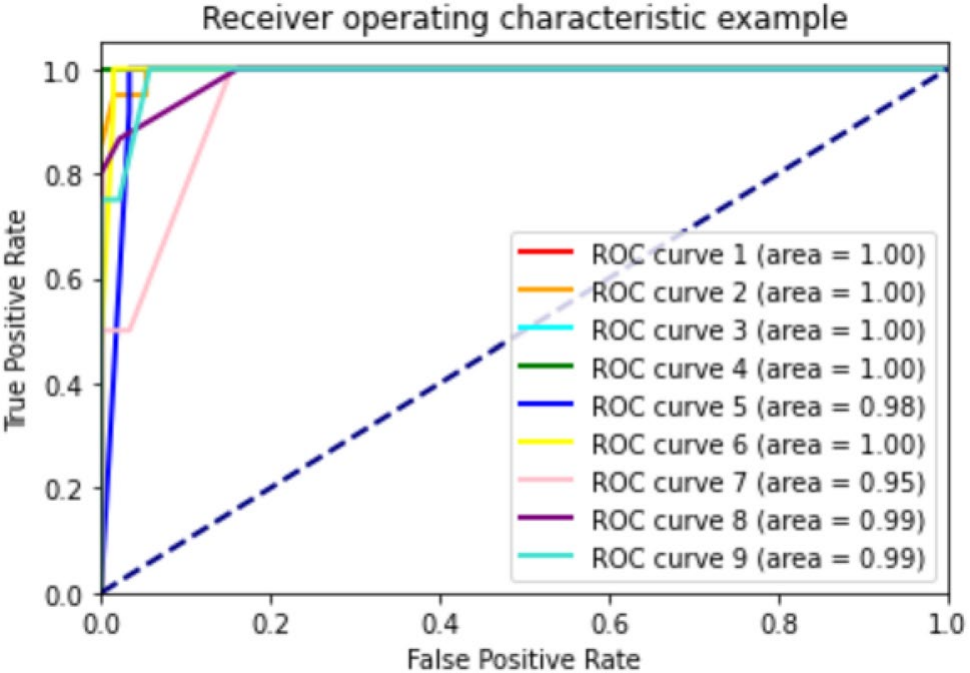
AUROC in RF algorithm for predicting exercise appropriate for healthy women and those with osteopenia and OP.

Based on the findings in Table 7, predicting exercise appropriate for healthy male individuals and those with osteopenia and osteoporosis, machine learning models achieved 56–84% accuracy, 45–78% precision, 50–69% sensitivity and 87–97% specificity. To predicting exercise appropriate for healthy female individuals and those with osteopenia and osteoporosis, machine learning models achieved an accuracy of 53–98%, a precision of 45–96%, a sensitivity of 37–97% and a specificity of 89–99%. The AB algorithm had the best performance in men and women (AUROC of 0.96, and 0.99, respectively). The ROC curves of different models of machine learning in men and women are presented in Figs. 4 and 5.

## Discussion

In the present study, various AI algorithms were used to predict and classify subjects into three categories of healthy, osteopenia and OP as well as to recommend appropriate sports programs. Accuracy was used to evaluate the prediction performance of algorithms.

In the first stage, The RF algorithm showed the highest performance in the men group with AUROC 0.91, and in the women's group, the GB algorithm with AUROC 0.95 performed best to identify healthy individuals, osteopenia and OP. Numerous studies have been previously carried out on diagnosing osteoporosis using AI.

A study published in 2016 by Yu et al.^31^ with a study sample of 119 hospitalized patients (49 men and 79 women) with average age of 65 years to diagnose osteoporosis using AI demonstrated that 55 patients had osteoporosis while 64 patients did not. In another study which targeted 1,792 postmenopausal women published in 2020 by Shim et al.^32^ compared seven machine learning models; the best performance was shown by the RF model with AUROC of 0.763. Ilio et al.^33^ used a set of 589 records extracted from the Greek population to measure bone density. In this study, three and five diagnostic factors were considered to predict the risk of osteoporosis, and finally, using multilayer perceptron classification, individuals were classified into three categories of normal, osteopenia and osteoporosis. In 2021, Wang et al.^34^ studied 1559 Chinese women over the age of 20 to develop an ANN model using age and weight as input for prediction of osteoporosis, achieving an AUROC of 0.78. A study in 2021 by Yang et al.^3^ entitled *Development of Machine Learning Models for Prediction of Osteoporosis from Clinical Health Examination Data* used a sample population of 3053 Taiwanese men and 2929 women; in this research, the best AUROC of 0.843 and 0.811 in men and in women, respectively was achieved with the RF algorithm for predicting osteoporosis. The following input characteristics were used: medical history of diabetes and hypertension, history of smoking and alcohol consumption liver function, thyroid function, lipid profile, blood protein content, electrolytes, hematological profile, renal function, and for women, history of obstetrics and gynecology were also included. Among male patients, secondary causes such as alcohol abuse, steroid therapy, and other metabolic disorders account for up to 65% of cases of osteoporosis^3^. In contrast, the prevalence of secondary osteoporosis is much lower in women than in men^35^. In women, estrogen deficiency after menopause and osteoporosis is one of the main causes of osteoporosis^36^. In the present study, for men and women, due to differences in cause and baseline characteristics, prediction algorithms were trained separately, obtaining acceptable results. Feature selection was based on accessibility and known relevance to bone health. In comparison with previous research, this had the benefits of predicting osteopenia and osteoporosis with a larger sample size selection (1224 patients) and the inclusion of more input features (17 features in men and 19 features in women) from different aspects. Reducing the number of variables (features) leads to poorer performance and the inclusion of more features contributes to better performance of AI models^3^. Input features were selected based on known association with bone health and easy availability.

Proposing appropriate and separate sport programs for healthy individuals and those with osteopenia and osteoporosis, the AB algorithm had the best performance in men and women (AUROC of 0.96, and 0.99, respectively).

To the best of our knowledge, the present research study is the first to use AI methods to recommend sports programs to individuals with osteoporosis and osteopenia. Age, and health status (healthy, osteopenia, and osteoporosis) were used as input to the algorithm, and appropriate exercise programs for improvement and treatment were selected based on effective and meaningful protocols mentioned in ISI authoritative articles. This research had the capability of recommending reliable exercise programs with different intensities and amounts based on patients' health status.

In the current study, accuracy was used to observe the performance of algorithms and categorize individuals into healthy, osteopenia, and osteoporosis categories, and accordingly propose appropriate exercise protocols. The great strengths of the study included the identification of a large number of risk factors directly associated with an increased risk of fracture. However, the present study has limitations such as removing inaccessible indicators of osteoporosis risk factors and not taking into consideration the alcohol consumption index because all recorded responses were negative. In addition, data from only three hospitals were included in this study and as such might not represent the entire population.

## Conclusion

Osteoporosis as a silent disease affects individuals worldwide and causes long-term loss of movement, serious injuries, severe pain, and even premature death. Using AI to predict risk groups can help the economy and reduce the burden on health systems. The results of our study showed that AI models with high accuracy and using several clinical and physiological indicators had acceptable performance in classifying individuals at risk for osteoporosis. Our study also demonstrated that AI algorithms have acceptable performance for accessing appropriate sport protocols, and by incorporating these algorithms into clinical practice, both physicians and patients can easily reap the benefits.

## Data Availability

The data supporting the findings of this study are available from the corresponding author upon reasonable request.

## Abbreviations

OP: Osteoporosis
AI: Artificial intelligence
DXA: Dual-energy x-ray absorption
BMD: Bone mineral density
PA: Physical activity
AUROC: Area under the receiver operating characteristic curve
DT: Decision tree
RF: Random forest
KNN: K-nearest neighbor
SVM: Support vector machine
GB: Gradient boosting
ET: Extra trees
AB: Ada Boost
ANN: Artificial neural networks
MLP: Multilayer perceptron
